# Toxicity of Usnic Acid: A Narrative Review

**DOI:** 10.1155/2022/8244340

**Published:** 2022-10-19

**Authors:** Nicoletta Croce, Michele Pitaro, Valentina Gallo, Giovanni Antonini

**Affiliations:** ^1^Interuniversity Consortium INBB-Biostructures and Biosystems National Institute, Rome 00136, Italy; ^2^Department of Sciences, University Roma Tre, Rome 00146, Italy

## Abstract

Usnic acid (UA) is a dibenzofuran derivative naturally present in lichens, organisms resulting from the symbiosis between a fungus and a cyanobacterium, or an alga. UA shows antimicrobial, antitumor, antioxidant, analgesic, anti-inflammatory as well as UV-protective activities. Its use as pharmacological agent is widely described in traditional medicine, and in the past few years, the product has been marketed as a food supplement for the induction of weight loss. However, the development of severe hepatotoxicity in a limited number of subjects prompted the FDA to issue a warning letter, which led to the withdrawal of the product from the market in November 2001. Data published in literature on UA toxicology, genotoxicity, mutagenesis, and teratogenicity have been reviewed, as well as the case reports of subjects who developed hepatotoxicity following oral administration of UA as a slimming agent. Finally, we reviewed the most recent studies on the topical use of UA, as well as studies aimed at improving UA pharmacologic activity and reducing toxicity. Indeed, advancements in this field of research could open the possibility to reintroduce the use of UA as therapeutical agent.

## 1. Introduction

**Figure 1:**
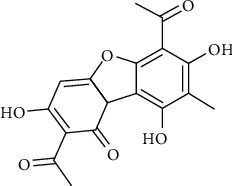
Molecular representation of UA.

Lichens are a form of symbiont between a fungus and an alga or cyanobacterium [1]. They contain a variety of organic compounds, some of them of pharmacological interest. Among these, usnic acid (UA) is a biologically active dibenzofuran derivative naturally present in several species of lichens such as *Alectoria, Cladonia, Usnea, Lecanora, Ramalina,* and *Evernia* (Figure 1) [2]. It comes as a bitter yellow powder, almost insoluble in water and in several organic solvents [3]. It is present in nature both in the dextrorotatory and in the levorotatory form, as well as a racemic compound [4].

Lichen extracts rich in UA have been used for a long time in traditional medicine [5]. The first documented use of UA dates back to the first century B.C. when extracts of dried lichens were officially included in the texts of traditional Chinese medicine. The product was used for the treatment of malaria, wounds, snakebite, and cough at the dose of 6–9 g of dried lichen taken in the form of tea or decoction, corresponding to 60–120 mg of UA per day [6].

UA shows a wide spectrum of biological and pharmaceuticals properties, including antibacterial, antiviral, antifungal, antiprotozoal, as well as antitumor activities. The antibacterial activity of UA has been the subject of several studies carried out after the end of the Second World War. However, the interest in the molecule gradually decreased in the 60s, due to the high costs of extraction from lichens, its poor solubility in water, and the development of chemically synthesized antibiotics. From the 80s onwards, UA has been the subject of a new wave of research since it could potentially represent an alternative to common antibiotics for the treatment of nosocomial infections linked to antibiotic resistance [7].

Studies carried out over the past thirty years have largely confirmed the efficacy of UA against different strains of bacteria. Studies focused on Gram-positive bacteria, such as different strains of staphylococci and enterococci, have shown that UA is more effective against the former compared to the latter [8, 9]. Overall, UA is effective against Gram-positive bacteria growing either in planktonic or in biofilm mode. For this reason, it could play a role in the management of biofilm-based wound infections [10].

Additional studies have been carried out with UA against different strains of mycobacteria. UA is effective against the human variant of *Mycobacterium tuberculosis*, as well as the bovine one. Interestingly, studies have demonstrated that UA maintains its antibacterial activity also against strains of *Mycobacterium tuberculosis* resistant to isoniazid, rifampicin, and streptomycin. These data confirm that UA could play a role in the fight against the increasingly widespread phenomenon of antibiotic resistance [11, 12].

The antiviral activity of UA has been successfully assessed against Epstein–Barr virus [13]. In association with zinc sulphate, UA turned out to be effective against genital papillomavirus infections [14]. Additional studies have shown that the UA exerts an effective antiviral activity against herpes simplex and poliovirus [15], as well as the H1N1 type A influenza virus [16].

UA has shown antifungal properties in combination with undecylenic acid for the treatment of *Tinea pedis* [17]. The compound is effective also for the treatment of protozoal diseases, such as cutaneous leishmaniasis [18]. Finally, the antitumor activity of UA has been demonstrated on in vitro models of hormone-dependent breast and prostate cancers. The results obtained so far confirm that the molecule is a potential antitumor agent for alternative cancer therapies [19].

Although the properties of UA are very interesting from a therapeutic point of view, its medical use is limited due to the low solubility of the molecule in water [20]. Furthermore, the use of high daily doses of UA orally taken for several days, often in association with other products for slimming purposes, resulted in 21 cases of hepatotoxicity documented by the Food and Drug Administration (FDA) in the 90s [21].

The main purpose of this review is to examine the data published so far on UA toxicology, genotoxicity, mutagenesis, and teratogenicity. On the basis of these data, case reports of hepatotoxicity in subjects who took food supplements containing UA for weight loss will be extensively reviewed. Finally, the possible use of UA through different routes of administration and the potential contribution of modern pharmaceutical techniques aimed at decreasing UA toxicity and increasing its bioavailability will also be examined.

## 2. Metabolism of UA

Data on absorption, distribution, metabolism, and excretion (ADME) of UA are limited. A study carried out in rabbit plasma and bovine serum albumin has shown that 99.2% of UA binds to plasma proteins [22]. Studies in rats treated with 25 mg/kg of UA administered intraperitoneally have demonstrated that the molecule is distributed in different tissues, with a higher concentration found in lungs and liver, followed by blood. The average tissue to plasma concentration ratio was 1.777, 1.503, and 1.192, respectively [22].

A pharmacokinetics study in rabbits treated with either 5 mg/kg intravenously or 20 mg/kg orally have shown UA half-lives of 10.7 ± 4.6 hours and 18.9 ± 2.9 hours, respectively [23]. The peak plasma concentration following oral administration was 32.5 ± 6.8 *μ*g/ml and it was reached after 12.2 ± 3.8 hours [24].

UA metabolism has been studied in plasma, hepatocytes, and subcellular fractions of human liver. UA was incubated in human liver S9 fractions, and samples were analysed by liquid chromatography/mass spectrometry (LC/MS). The hepatic clearance of UA was 13.86 ml/min/kg. This study showed that UA is metabolized by the 1A2 isoform of cytochrome P450 (CYP1A2) since furafylline (a specific inhibitor) increased the half-life of UA by tenfold. On the other hand, conjugation of UA with glycuronic acid depends on two isoforms of uridina diphosphate-glycuronosyl transferase (UGT), namely, 1A1 and 1A3. The combined activity of CYP1A2, UGT1A1, and UGT1A3 produce three different monohydroxylated metabolites and two regioisomeric glucuronide conjugates of the parent drug [25].

## 3. Toxicity of UA In Vitro

Several studies aimed at identifying the molecular basis of UA hepatotoxicity have been carried out in human hepatoblastoma cell lines, as well as in primary hepatocyte cultures. Murine hepatocytes were cultured by Han et al. in a medium added with 5 *μ*M of UA [26]. After 16 hours, 98% of cells were necrotic, without any sign of apoptosis. UA induced a significant decrease in the ATP level due to the uncoupling of oxidative phosphorylation. Oxidative stress seemed to play a key role, since

- Pretreatment with antioxidants (hydroxytoluenebutylate and vitamin E) reduced UA-induced necrosis by approximately 70%
- Mitochondrial glutathione (GSH) depletion with diethylmaleate increased hepatocyte susceptibility to UA
- UA treatment increased free radical production

UA autoxidation and increased hydrogen peroxide production in mitochondria were responsible for the increased level of reactive oxygen species following the addition of UA to the medium. At the concentrations tested, UA was hepatotoxic, triggered oxidative stress, and disrupted the normal metabolic pathways [26].

Pramyothin et al. evaluated the hepatotoxic effects of UA in rats, isolated rat hepatocytes, and isolated rat liver mitochondria [27]. Following the treatment of rats with 200 mg/kg of the dextrorotatory enantiomer of UA administered intraperitoneally for 5 days, no significant changes in serum transaminase levels were observed. However, electron microscopy showed some morphological changes in mitochondria and endoplasmic reticulum. Isolated rat hepatocytes cultured in a medium containing UA 1 mM showed signs of cell membrane damage. The toxic effect was similar to the reference hepatotoxin (i.e., carbon tetrachloride). Increased level of transaminases, lipid peroxidation, and CYP 2E1 activity were observed, with a concomitant decrease of GSH level. UA at a concentration between 0.15 and 6 *μ*M uncoupled the oxidative phosphorylation in mitochondria isolated from hepatocytes. In summary, high concentrations of UA induced loss of membrane integrity by impairing mitochondrial respiration and oxidative phosphorylation [27].

The aforementioned study by Foti et al. showed that UA at a concentration of 20 *μ*M does not induce CYP1A2, CYP2B6, or CYP3A4 in human hepatocytes [25]. UA is a potent inhibitor of CYP2C19 (IC50 = 9 nM) and CYP2C9 (IC50 = 94 nM). However, preincubation of microsomes with UA did not induce a significant inhibition of CYP2C19. Data *in vitro* suggest that UA might potentiate hepatotoxicity induced by other drugs. However, the development of hepatotoxicity depends also on other cofactors, such as the induction of CYP1A, the coadministration of CYP1A2 inhibitors, the presence of specific UGT1A1 polymorphisms, the presence of hyperbilirubinemia, or the coadministration of CYP2C substrates with low therapeutic index [25].

Sonko et al. published a paper on UA toxicity in primary rat hepatocytes. In order to better understand the metabolic pathways involved in the development of cell toxicity, the authors assessed cell viability, ATP concentration and ^13^C isomer distribution. Cells were exposed to UA concentrations of 0, 1, 5, or 10 *μ*M for a time course of 2, 6, or 24 hours. At the end of the treatment, aliquots of supernatant were collected to determine the distribution of the ^13^C isomer isotope in CO_2_, lactate, glucose, and glutamate. The 1 *μ*M concentration of UA did not produce significant changes in cell viability compared to controls. However, 5 and 10 *μ*M concentrations significantly decreased cell viability with increasing exposure times. Overlapping results were obtained in ATP depletion experiments. The 1 and 5 *μ*M concentrations increased oxidative phosphorylation, whereas the 10 *μ*M concentration produced a significant inhibition of oxidative phosphorylation and gluconeogenesis. The increase in oxidative phosphorylation observed at the lower concentration could be an adaptive response to compensate the decreased mitochondrial function [28].

Sahu et al. evaluated the metabolism and toxicity of UA in human hepatoblastoma cell line G2 (HepG2). Cells were treated with control vehicle alone or with UA at concentrations ranging from 0 to 100 *μ*M for 24 h. At the end of the treatment period, cytochrome P450 activity, cytotoxicity, oxidative stress, mitochondrial dysfunction, and changes in gene expression profiles were assessed. Exposure to UA increased the cytochrome P450 activity, cytotoxicity, oxidative stress, and mitochondrial dysfunction. UA significantly modified the expression of six of the 84 genes examined. In particular, it was observed an increased activity of C-C motif chemokine ligand 21 (CCL21), CCNC and UGT1A4, genes associated with inflammation, cell proliferation, and DNA damage and repairing processes, respectively. On the other hand, it was observed a decreased activity of colony stimulating factor 2 (CSF2), CYP7A1 and CYP2E1, genes associated with inflammation and oxidative stress, respectively. In summary, the biomarkers assessed confirmed UA toxicity in HepG2 cells, suggesting an oxidative mechanism of action [29].

A second study by Sahu et al. assessed the interaction between UA and lipopolysaccharide in hepatotoxicity induction [30]. The rationale for the study lays in the fact that lipopolysaccharide may represent a contaminant of food. HepG2 cells were treated with UA, lipopolysaccharide, and control vehicle, either separately or in combination, for 24 hours at 37°C in 5% CO_2_. After the treatment, cells were evaluated by analysis of traditional biochemical endpoints of toxicity in combination with toxicogenomics endpoints (cytotoxicity, oxidative stress, mitochondrial damage, and changes in gene expression patterns). Taken individually, low concentrations of UA and lipopolysaccharide did not affect cell viability. On the other hand, their association increased cytotoxicity, oxidative stress, and mitochondrial damage. Gene expression patterns analysis showed significant changes of several genes. Study results suggest some interaction between UA (at non-toxic concentrations) and lipopolysaccharide [30].

Shi et al. studied the toxicity of UA in primary cultures of rat hepatocytes. Inhibition of CYP activity with SKF-525A (20 *μ*M), alpha-naphthoflavone (10 *μ*M), or ketoconazole (25 *μ*M) significantly increased the toxicity of UA at concentrations between 3 and 6 *μ*M after 3 to 20 hours, as demonstrated by the release of lactate dehydrogenase (LDH) from the cells. Two hours after UA exposure and before LDH release from cells, CYP inhibitors potentiated the inhibition of cellular respiration and ATP depletion. These data demonstrate that UA is transformed in rat hepatocytes into less toxic metabolites, probably through the action of CYP1A and CYP3A [31].

A study by Chen et al. investigated the mechanisms underlying the hepatotoxicity of UA and the role played by autophagy. Studying HepG2 cells, the authors observed that UA induces apoptosis, confirmed by increased caspase-3/7 activity and increased subdiploid core formation. On the other hand, UA-induced autophagy was demonstrated by conversion of LC3B-I to LC3B-II and degradation of P62. Treatment of cells with autophagy inhibitors (3-methyladenine or chloroquine) or small interfering RNAs against autophagy-related 7 (Atg7) increased apoptosis and reduced cell viability, demonstrating that autophagy protects cells from UA-induced toxicity. The molecule activates the mitogen-activated protein kinase (MAPK) pathway, whereas suppression of JNKs with a specific inhibitor increases apoptosis by reducing autophagy. In turn, inhibition of autophagy reduced JNK activity. As a matter of facts, UA modifies cytoplasmic signals, and the induction of autophagy seems to be a defensive mechanism against molecule-induced toxicity [32].

A second study by Chen et al. evaluated the effects of UA on the endoplasmic reticulum of Hep2 cells. The authors observed that UA induces stress in the endoplasmic reticulum, as demonstrated by the increased expression of markers such as C/EBP homologous protein (CHOP), activating transcription factor 4 (ATF-4), phosphorylation of eukaryotic initiation factor-2*α* (p-eIF2*α*), and *X*-box binding protein 1 (XBP1). In addition, the endoplasmic reticulum stress inhibitor 4-phenylbutyrate attenuated UA-induced apoptosis. UA significantly increased free calcium in the cytosol, the expression of calcium release-activated calcium channel protein 1 (ORAI1) and stromal interaction molecule 1 (SIM1), two key components of store-operated calcium entry (SOCE) that represents the main calcium transport system in nonexcitable cells. Furthermore, knockdown of ORAI1 prevented endoplasmic reticulum stress and ATP depletion in response to UA. Nevertheless, overexpression of ORAI1 increased endoplasmic reticulum stress and ATP depletion caused by UA. Overall, UA interferes with calcium homeostasis and induces stress in the endoplasmic reticulum. UA-induced cellular damage is produced, at least partially, by the activation of SOCE calcium channels [33].

A third study by Chen et al. showed that UA activates nuclear factor erythroid 2-related factor 2 (Nrf2) and increases the activity of c-Jun N-terminal kinases (JNKs) and Akt/mammalian target of rapamycin (mTOR). HepG2 cells were plated at a concentration of 3 × 10^5^ cells/ml in 96-well multiwells. Cells were cultured for 24 hours before treatment with either 0.1% UA or vehicle alone (DMSO). The authors observed that UA-induced DNA damage and cell cycle arrest in S-phase. UA-induced oxidative stress, as demonstrated by the production of reactive oxygen species (ROS) and GSH depletion. Exposure for 6 hours to UA significantly increased Nrf2 level, promoted its translocation into the nucleus, increased the expression of proteins related to the antioxidant response, and induced the expression of Chen2-regulated targets such as glutathione reductase, glutathione S transferase, and NAD(P)H quinone oxidoreductase-1 (NQO1). Knockdown of Nrf2 with shRNA enhanced UA-induced damage and cytotoxicity. These data demonstrate that UA produces dysregulation of cell cycle, DNA damage, and oxidative stress, and UA-induced cytotoxicity activates the transcription factor Nrf2 [34].

Piska et al. studied the biotransformation of UA enantiomers into reactive products using a GSH assay in human, mouse, and rat microsomes. The results of the study showed that each enantiomer produces two reactive glutathione-binding metabolites, which could at least partially explain the toxicity of UA [35].

A recent study by Kwong et al. focused on the role played by porimin in UA-induced hepatotoxicity by several techniques (immunoblotting, siRNA transfection to induce knockdown of specific genes etc) using both normal human L02 cells and ICR mice. Assays were performed to assess oxidative stress, GSH, malondialdehyde (MDA), and superoxide dismutase (SOD) levels. Identification of apoptotic cells was performed by double fluorescence. Expression of proteins such as glutathione S transferase, glutathione reductase, glutathione peroxidase 4, catalase, N-terminal kinase-Jun, caspases, gastamin-D, and porimin was assessed by Western blot. Exposure to UA induced a significant increase in the expression of glutathione reductase, glutathione S transferase, and glutathione peroxidase-4 in L02 cells, while the expression of catalase was decreased in a dose-dependent manner. The dextrorotatory isomer of UA did not activate caspase-3, caspase-1, or gasdermine-D, but significantly increased the expression of porimin. The dextrorotatory isomer of UA did not cause cytotoxicity in L02 cells silenced for porimin. The authors conclude that increased porimin expression and pore formation in the membrane could contribute to UA-induced hepatotoxicity [36].

In summary, the two main mechanisms of UA toxicity seem to be the uncoupling of oxidative phosphorylation and free radical generation. The former decreases ATP synthesis, and the latter induces cell membrane and mitochondrial injury, with a mechanism similar to that observed following the administration of carbon tetrachloride. The main outcomes of the *in vitro* studies carried out on UA toxicity are summarized in Table 1.

## 4. Toxicity of UA In Vivo

Several studies have been carried out to assess UA toxicity in vivo. The study by Pramyothin et al. has been already described in Section 3 since it included tests in vitro, as well as in vivo [27].

Joseph et al. examined the expression levels of 542 genes in the liver of B6C3F female mice using a mitochondria-specific microarray. Mice received UA at 0, 60, 180, and 600 ppm in their diet for two weeks. Microarray analysis showed a significant induction of genes associated with complexes I through IV of the electron transport chain only at the highest dose of 600 ppm. Several genes involved in fatty acid oxidation, Krebs cycle, apoptosis, and membrane transporters were also overexpressed. The authors argued that the upregulation of complexes I–IV may be a compensatory mechanism to maintain the proton gradient across the mitochondrial inner membrane, while induction of fatty acid oxidation and the Krebs cycle may be an adaptive response to uncoupling of mitochondria [37].

Lu et al. studied the hepatotoxicity of the dextrorotatory enantiomer of UA on rats by mass spectrometry: Wistar rats were treated with three different doses of UA: 100, 200, and 240 mg/kg daily for 8 days. The main biochemical and histopathological parameters were assessed together with a metabolomic analysis performed by gas chromatography/mass spectrometry. The ratio of liver/body weight was significantly increased in UA-treated rats, as well as transaminase and total bilirubin levels. Signs of diffuse hepatocyte degeneration were observed in liver sections of rats treated with the higher dosages of UA. Exposure of rats to UA-induced changes in energy, amino acid, lipid, and nucleotide metabolism [38].

Liu et al. studied the proteome expression in UA-induced hepatotoxicity in rats: expression profiles were analysed by two-dimensional gel electrophoresis followed by mass spectrometry. The results showed significant changes in the expression of ten proteins associated with oxidative stress and lipid metabolism, suggesting that endoplasmic reticulum and mitochondria might be the primary targets in UA-induced hepatotoxicity [39].

Moreira et al. studied the hepatotoxicity of UA in livers of male Wistar rats perfused in a system without recirculation [40]. At low concentrations, UA stimulated oxygen consumption, decreased intracellular ATP levels, increased the NADH/NAD + ratio in the cytosol, while decreasing it in the mitochondria, strongly inhibited gluconeogenesis from three different substrates (IC50 between 1.33 and 3.61 *μ*M), and stimulated glycolysis and glycogenolysis. UA increased the production of 14 CO_2_ from ^1–14^C octanoate and ^1–14^C oleate, but ketogenesis from octanoate was reduced (no differences were observed in ketogenesis from oleate). The authors conclude that, up to a concentration of 2.5 *μ*M, UA is an uncoupler of oxidative phosphorylation. At higher concentrations, other effects occur, namely, inhibition of electron flow in mitochondria and inhibition of medium-chain fatty acid oxidation. From a metabolic point of view, it is possible to predict a more pronounced UA toxicity during fasting due to the decreased hepatic flux of glucose and ketone bodies [40].

In summary, data from *in vivo* studies confirm those observed in *in vitro* studies and show that UA, usually at doses equal or higher than 200 mg/kg/day, is an uncoupler of oxidative phosphorylation. The main outcomes of the *in vivo* studies carried out on UA toxicity are summarized in Table 2.

## 5. Genotoxicity, Mutagenesis, and Teratogenicity

Leandro et al. studied the genotoxicity of UA in vitro on V79 cells and in vivo on Swiss mice. In this study, V79 cells were exposed to UA concentrations of 15, 30, 60, and 120 *μ*g/ml. Swiss mice were treated with UA at doses of 25, 50, 100, and 200 mg/kg body weight. The same concentrations of UA were combined with methyl-methanesulfonate (MMS) to assess antigenotoxicity. In vitro studies showed that UA damages DNA at concentrations of 60 and 120 *μ*g/ml in the Comet assay, whereas no genotoxic effect was observed in the micronucleus assay. In vivo, no genotoxic effect was observed, and combined administration of UA and MMS significantly decreased the frequency of micronuclei and DNA damage both in vitro and in vivo. Although the mechanisms underlying the protective effect demonstrated by UA are not completely understood, the antioxidant activity of the molecule could explain the protective effect against MMS-induced genotoxicity [41].

Koparal et al. evaluated the potential genotoxicity of UA on V79 (Chinese hamster lung fibroblasts), A549 (human lung carcinoma cells), and human lymphocytes cell lines by MTT and CBMN assays. The results of the study showed that both enantiomers of UA lacked genotoxicity but induced a significant cytotoxic and proapoptotic effect in tumour cell lines [42].

The genotoxicity of UA was the subject of a study by Polat et al. carried out on human lymphocytes exposed to UA at concentrations ranging from 0 to 200 *μ*g/ml. Genotoxic damage was evaluated by chromosomal and micronucleus aberration assays. Furthermore, biochemical parameters, such as total antioxidant capacity and total oxidative status, were evaluated. The authors demonstrated that UA has no mutagenic effects on human lymphocytes. Overall, the results obtained indicate that low concentrations of UA (1 and 5 *μ*g/ml) increase the total antioxidant capacity in cultured human cells and that the total oxidative status is maintained unchanged [43].

Machado et al. evaluated the mutagenic potential of the dextrorotatory enantiomer of UA by SMART (somatic mutation and recombination test) assay to detect epithelial tumour clones in *Drosophila melanogaster*. For this purpose, 72 ± 4 hr *Drosophila* larvae were treated with UA (5, 10 or 20 mM) and urethane (10 mM) as positive control or solvent (Milli-Q water, 1% Tween-80 and 3% ethanol as negative control). The results of the study indicate that, at the doses tested, UA can exert mutagenic effects on *Drosophila melanogaster* somatic cells [44].

A study by Silva et al. focused on the potential teratogenic effects of UA. Female rats were randomized into three study groups and treated with (a) 25 mg/kg body weight of UA; (b) 15 mg/kg body weight of UA; and (c) control treated with vehicle only. Treatments were administered with drinking water between days 6 and 15 of pregnancy, and after 20 days, the foetuses were removed and analysed. Low weight gain, increased resorption, and reduced number and weight of viable foetuses were found. Morphologic changes ranging from eye exposure to limb atrophy were found at the highest dose. From a histological point of view, in the group treated with the highest dose of UA, fewer megakaryocytes and more hepatocytes were observed in the liver of the foetuses. The experimental model used suggests the possibility of teratogenic effects during organogenesis in the foetus [45].

Prokopiev et al. studied the genotoxic effects of dextrorotatory and levorotatory enantiomers of UA on human mononuclear cells: the enantiomers of UA at concentrations between 0.04 and 0.30 mM induce a genotoxic effect. In this study, the genotoxicity of the levorotatory enantiomer was twice as high as that of the dextrorotatory enantiomer [46].

In a second study, Prokopiev et al. evaluated the genotoxic effects of dextrorotatory and levorotatory enantiomers of UA in murine liver and kidney cells. DNA damage was observed 1 hour after UA administration at doses ranging from 100 to 50 mg/kg. Genotoxic damage is associated with oxidative stress in cells, with no significant difference between dextrorotatory and levorotatory enantiomer [47].

## 6. Myocardiotoxicity

High doses of the dextrorotatory isomer of UA produce uncoupling of oxidative phosphorylation and subsequent stress in mitochondria and endoplasmic reticulum. This mechanism appears to underlie the hepatotoxic effects observed when the product is administered orally at high doses. However, it is unclear whether UA induces toxicity to other mitochondria-rich organs such as the heart.

Yokouchi et al. randomized 27 female rats into three study groups of 9 animals each treated with (a) 30 mg/kg body weight of UA; (b) 100 mg/kg body weight of UA; (c) vehicle alone (0.5% methylcellulose solution) [48]. The treatment administered orally once daily for 14 consecutive days did not change hematochemical parameters. However, the higher dose caused swelling of mitochondria and increased expression of prohibitin in the sarcoplasmic reticulum, with toxicogenomic analysis showing increased expression of genes related to oxidative stress. It should be noted that a dose of 100 mg/kg of body weight corresponds to 7 g in a person with a body weight of 70 kg [48].

Cheng et al. evaluated the acute toxicity of the dextrorotatory isomer of UA in mice and rat cardiac fibroblasts. The LD50 of the dextrorotatory isomer of UA in mice after oral administration was 388 mg/kg. The IC50 in rat cardiac fibroblasts was 322 *μ*g/ml. The authors conclude that the dextrorotatory isomer of UA is a naturally occurring compound with low toxicity in mice and it does not produce obvious toxic effects when administered at medium and low doses [49].

## 7. Neurotoxicity

The interactions of UA with genes, proteins, and other molecules that play a key role in cellular oxidoreductive balance and nitric oxide production were assessed by Rabelo et al. [50]. The study also evaluated the oxidoreductive properties of UA against several reactive oxygen species generated in vitro. Since no data have been previously published on the *in vitro* neurotoxicological effects of UA, the authors evaluated its effects on neuron-like cells named SH-SY5Y. At the highest concentrations tested, UA showed significant antioxidant activity evaluated as the total reactive antioxidant potential (TRAP) index. In addition, UA was also effective against hydroxyl radicals and decreased the nitric oxide production. The MTT reduction assay demonstrated increased *in vitro* lipoperoxidation and modified cell viability at the highest concentration of 20 *μ*g/mL for 1 and 4 hours, as well as at 2 and 20 *μ*g/mL for 24 hours. UA showed no protective effects against hydrogen peroxide-induced cellular damage. The production of reactive oxygen species assessed by DCFH assay showed that UA induces changes in their basal production at concentrations of 20 *μ*g/ml for 1 h and from 2 to 20 *μ*g/ml for 4 and 24 h. In summary, UA shows different oxidoreductive properties depending on the conditions and cellular environments tested. The study also suggests that the potentially neurotoxic effects of UA should be investigated by further approaches, i.e., by *in vivo* studies and clinical trials [50].

## 8. Toxicity of UA Used Orally as a Slimming Agent

The aforementioned *in vitro* and *in vivo* studies clearly demonstrate that UA is an uncoupler of oxidative phosphorylation. Since UA decreases the efficiency of energy use and increases thermogenesis, it has been included in the composition of several food supplements intended for the induction of weight loss in obese subjects. Two of these, namely, Lipokinetix (Syntrax, Cape Girardeau, Missouri, US) and UCP-1 (BDC Nutrition, Richmond, KY) have been marketed in the US until 2001 [51]. Each capsule of Lipokinetix contained sodium usniate (100 mg), norephedrine (25 mg), diiodothyronine (100 mg), yohimbine (3 mg), and caffeine (100 mg) [51]. The recommended dose of LipoKinetix was 2 capsules 3 times per day, corresponding to an overall UA daily intake of 600 mg. Regarding UCP-1, each capsule of the product contained 150 mg of UA, 525 mg of L-carnitine and 1050 mg of calcium pyruvate. The recommended dose of UCP-1 was 3 capsules three time per day, corresponding to an overall UA daily intake of 1350 mg [51].

Overall, Lipokinetix and UCP-1 were responsible for at least 21 cases of hepatotoxicity reported to the FDA. Among these, one subject died, one underwent a liver transplant, seven developed acute liver failure, ten experienced elevated transaminases, and four developed mild hepatotoxicity. On the basis of these findings, the FDA issued a warning letter, and the products were withdrawn from the market in November 2001. Several cases of UA-induced hepatotoxicity have been published in literature.

Favreau et al. described five cases of Japanese nationals residing in the Los Angeles area treated for acute liver failure between July and December 2000 at the Cedars-Sinai Medical Center (Los Angeles, CA) [52]. Two more cases of white bodybuilders were identified through the FDA Med Watch program. All subject took Lipokinetix according to the manufacturer's recommendations. Interestingly, none of these subjects was obese, confirming that, in most cases, people take food supplements without any clear indication. Three patients developed jaundice, and one developed fulminant hepatic failure within 4–12 weeks after treatment initiation. All patients recovered, and their hematochemical parameters returned to normal within four months in five patients (the other two refused to undergo further examination). Two patients resumed food supplements other than LipoKinetix without any other problem [52].

In twenty cases of acute liver failure published by the Oregon Health & Science University Hospital and the Portland Veterans Affairs Medical Center in Oregon, ten were regular users of dietary supplements and herbal products. In two of these cases (a 25-year-old woman who died and a 42-year-old man who recovered), Lipokinetix was identified as the sole cause of acute liver failure [53].

A retrospective analysis has been carried out on 12 patients who developed hepatotoxicity related to the ingestion of herbal products for the induction of weight loss. Two out of the 12 patients reviewed took Lipokinetix according to the manufacturer's recommendations. One patient recovered, while the other one underwent liver transplantation [54].

Durazo et al. described the case of a 28-year-old woman taking 500 mg of UA (Pure Usnic Acid, Industrial Strength AAA Services, Frazer Park CA) daily as slimming agent. She stopped the treatment after two weeks because she began to feel sick. After two weeks, she resumed the treatment for 4 days. Due to the development of signs and symptoms of hepatotoxicity, she was transferred to the Medical Center of the University of California, Los Angeles (UCLA), where she underwent liver transplant. She recovered completely, and the examination of the injured liver demonstrated a shrunken organ (470 g, while the weight of a normal liver is usually around 1,200 g) with scattered irregularly shaped nodules of residual parenchyma [55].

Sanchez et al. described two subjects who developed severe hepatotoxicity while using UCP-1 at the recommended daily dose. The first one was a 38-year-old female fitness instructor who developed asthenia, abdominal pain, and jaundice after three months of treatment. The patient was taking oral contraceptives, but never had liver disease in the past, was not an alcohol abuser, and was not at risk of developing viral hepatitis. Laboratory tests showed elevated levels of bilirubin, alkaline phosphatase, aspartate aminotransferase, and alanine aminotransferase. Patient's condition progressively worsened, with the development of grade 2-3 hepatic encephalopathy. The patient underwent liver transplantation, and the explant pathology revealed a shrunken liver with diffuse parenchymal necrosis. The patient recovered rapidly and was discharged a week after the surgery intervention. Her husband was also taking UCP-1 with the same schedule of administration. He was asymptomatic; however, hematochemical examinations showed a marked increase in transaminases, which returned to basal four months after stopping the treatment [56].

Krishna et al. described the case of a 28-year-old female bodybuilder treated at the Mount Sinai Medical Center (New York, NY) for progressive malaise and jaundice. The patient took two food supplements, namely, Somalyz and Lipolyz (Species Nutrition, USA) containing 4 mg and 12 mg of UA per capsule, respectively. She was taking 1-2 capsules of Somalyz at bedtime and one capsule of Lipolyz three times per day with meals, corresponding to an overall daily UA intake of 44 mg for one month before symptoms onset. The patient underwent successful liver transplantation. Gross examination showed massive necrosis of the liver and parenchymal collapse [57].

## 9. Topical Use of UA

Topical application of lichen extracts containing UA has been used in folk medicine for wound healing, athlete's foot, and sore throat [51]. UA is currently used in industry to produce perfumes, cosmetics, and medical ointments [58], as well as toothpaste, mouthwash, deodorants, and sunscreen products, either as active principle or preservative [59]. UA has been successfully used in combination with zinc sulphate for the treatment of genital papillomavirus infections [14].

Recently, Zhang et al. investigated the activity of UA applied topically in wound healing. The effects were measured by means of wound contraction experiments, histological analysis, and immunohistochemistry. Study results showed that wound healing rates were higher and re-epithelialization time shorter following topical application of UA compared to control. Treatment with UA decreased the number of inflammatory cells, while increasing fibroblast proliferation, granulation tissue, and vascular regeneration. UA-induced earlier complete re-epithelialization, production of well-organized bands of collagen, and epidermal keratinization. The authors conclude that the topical use of UA promotes wound healing thanks to the anti-inflammatory properties of the molecule [60]. Several wound dressings based on UA have been tested in the last few years in vitro, as well as in animal models of dermal burns [61].

Coşkunmeriç et al. developed an innovative formulation of UA in nanogel for the treatment of oral ulcers. UA in nanogel accelerated wound healing in an animal model and showed an effective antimicrobial activity against Bacillus Cereus [62].

Galanty et al. compared the photoprotective properties of the two UA enantiomers. Both the compounds showed good skin-penetrating properties; however, the left-handed enantiomer was slightly more toxic to keratinocytes compared to the right-handed one. On the other hand, (+)-UA showed good photoprotective properties and photostability, comparable to the UV filter octocrylene. Surprisingly, (+)-UA in combination with octocrylene showed enhanced photoprotective properties and improved photostability. The authors concluded that (+)-UA can be used in cosmetic products as a UV filter [63].

Recently, the ENEA Casaccia Research Center (Rome, Italy) carried out a study on 30 Wistar rats randomized to receive two nasal spray formulations containing UA at the concentration of 75 *μ*g/ml or 150 *μ*g/ml (personal communication by Dr. Maria Teresa Mancuso). The spray is a medical device intended for the prevention of airborne viral infections. Rats were treated with 30 *μ*l per nostril 3 times daily for one week. At the end of the treatment period, no signs of inflammation were observed in the nasal mucosa, as well as in the liver, kidney, spleen, lungs, brain, and heart. Serum aspartate aminotransferase (AST) and alanine aminotransferase (ALT) levels were in the normal range, and UA serum concentration measured by LC/MS was very low (around 0–6 *μ*g/ml).

Overall, topical application of UA appears to be safe and well tolerated.

## 10. Encapsulation of UA in Nano- and Microparticles

Due to the numerous biological and therapeutical properties of UA, several studies investigated many strategies to improve its pharmacologic activity and reduce toxicity, including the use of diverse drug delivery systems. In particular, the encapsulation of UA into nano- and microcarriers, including liposomes and nanoemulsion, was considered. Indeed, besides enhancing drug stability, solubility, and intracellular uptake, these delivery systems can also grant a sustained and controlled release of the drug, thus modulating toxicity and favouring drug accumulation in the target tissues.

Diverse in vitro studies reported the successful encapsulation of UA into liposomes, nanoemulsions, polymeric nanospheres, and polymeric or magnetic nanoparticles and demonstrated the positive impact of encapsulation on the enhancement of UA properties, especially focussing on antitumoral, antimicrobial, and antioxidant activities. In parallel, these studies also investigated the toxicity reduction of encapsulated UA [64–66].

Lira et al. were the first who studied the in vitro antimicrobial effects of UA encapsulated into liposomes. Authors demonstrated the enhanced antibacterial activity of UA-loaded liposomes on *Mycobacterium tuberculosis*, compared to free UA and suggested their potential in targeting *M. tuberculosis*-infected macrophages for the treatment of pulmonary tuberculosis [67]. Subsequently, these findings were confirmed by studies on multidrug-resistant tuberculosis clinical isolates [65]. These results were recently improved by Saviano et al. that described an ameliorated formulation of encapsulated UA, based on fucoidan-coated liposomes [68]. In addition, also other studies reported similar results for different bacterial strains, including methicillin-resistant *Staphylococcus aureus* [69] and *S. epidermidis* [70].

Several studies also evaluated the antitumoral activity of encapsuled UA [66]. One of these demonstrated that UA encapsulated into PLGA-microspheres promoted an increase of 21% in the tumour inhibition as compared with free UA [71]. Another study demonstrated the potentiated attenuation of skin carcinogenicity, in mouse models, after the treatment with UA blended nanoemulsions, compared to free UA [72]. In addition, other authors studied the in vivo antitumor activity of UA-loaded nanocapsules in male mice with Sarcoma-180 tumour. Their results showed a significative reduction in tumour growth compared to controls with a 26% increase of antitumor activity of encapsulated UA [73].

The effects of UA nanoencapsulation have been also assessed for the evaluation of toxicity both in in vivo and in vitro studies. In this context, results were controversial. Some authors reported no significative changes in encapsulated UA toxicity, compared to free UA, and some studies reported even an increase of the UA toxicity after encapsulation [67, 74]. On the other hand, other studies reported a significative reduction of toxicity. In a study, UA hepatotoxicity was shown to be substantially reduced in in vivo studies when animals were treated with UA-loaded nanocapsules [73]. In another study, the toxicity of encapsulated UA was evaluated in vitro on HEp-2 cell lines using the MTT method, confirming a reduction of toxicity, compared to free UA [71]. Other authors confirmed these results, including a study that, besides assessing an increase of the anti-inflammatory effects of UA-loaded polymeric microparticles, and also reported a marked reduction of acute toxicity [75].

Overall, the results obtained in these studies are promising and could lead to extend the use of UA also for systemic administration, opening the possibility of exploiting the UA properties for the therapy of various diseases, including infectious, tumoral, and inflammatory. However, although several studies have fully demonstrated the enhancement of UA therapeutical properties after encapsulation, further studies are needed to validate the in vivo effectiveness of encapsulation in reducing UA toxicity.

## 11. Conclusions

UA is a promising therapeutic agent with antibacterial, antiviral, antifungal, antiprotozoal, and antitumor activities. Lichen extracts rich in UA have been used in traditional medicine for the treatment of malaria, wounds, snakebite, and cough. The molecule is currently used in industry to produce perfumes, cosmetics, and medical ointments, as well as toothpaste, mouthwash, deodorants, and sunscreen products.

Intake of food supplements containing high doses of UA (usually ranging from 600 to 1,350 mg/day) for slimming purposes resulted in 21 cases of hepatotoxicity documented by the FDA. Several studies *in vitro* and *in vivo* confirmed that high concentrations of UA are hepatotoxic, due to the uncoupling of oxidative phosphorylation and free radical generation. The molecule is not genotoxic; however, data on mutagenesis and teratogenicity are quite controversial.

On the other hand, topical use of UA seems to be safe and well tolerated. The product could be potentially used in a wide range of applications, ranging from bioactive wound dressing to nasal spray for the prevention of airborne infections. UA encapsulation in micro- and nanocarriers could potentially improve UA bioavailability and decrease its toxicity. However, further studies are needed to confirm these preliminary observations.

## Figures and Tables

**Table 1:** Main outcomes of the *in vitro* studies on UA toxicity.

Reference	Model	UA concentration	Main outcomes
Han et al. [26]	Murine hepatocytes	5 *μ*M	Uncoupling of mitochondrial electron transportation chain
			Decrease of ATP level
			Increase of ROS

Pramyothin et al. [27]	Rat hepatocytes	1 mM	Damage of cell membranes
			Lipid peroxidation
			Induction of CYP2E1 activity
			Decrease of GSH level
	Rat liver mitochondria	0.15–6 *μ*M	Uncoupling of oxidative phosphorylation

Foti et al. [25]	Human hepatocytes	20 *μ*M	Inhibition of CYP2C19 and CYP2C9

Sonko et al. [28]	Rat hepatocytes	1 *μ*M	No changes in cell viability
			Increase of oxidative phosphorylation
		10 *μ*M	Decreased cell viability
			Inhibition of oxidative phosphorylation
			Decrease of gluconeogenesis

Sahu et al. [29, 30]	HepG2 cells	0–100 *μ*M	Increase of CYP activity
			Increase of oxidative stress and mitochondrial dysfunction
			Changes in gene expression
			Possible interaction with LPS in toxicity induction

Shi et al. [31]	Rat hepatocytes	3–6 *μ*M	UA toxicity increased by CYP inhibitors

Chen et al. [32–34]	HepG2 cells	3.13, 12.5, 50 *μ*M	Induction of autophagy and apoptosis
			Stress of the endoplasmic reticulum
			Activation of Nrf2

Piska et al. [35]	Human, rat and mouse microsomes		Production of two GSH-binding metabolites probably responsible of hepatotoxicity

Kwong et al. [36]	Human L02 cells		Hepatotoxicity possibly related to increased level of porimin

**Table 2:** Main outcomes of the *in vivo* studies on UA toxicity.

Pramyothin et al. [27]	Wistar albino rats	50 or 200 mg/kg for 5 days	No changes in transaminase level
			Mitochondria and ER damage with the highest dose of UA

Joseph et al. [37]	B6C3F mice	0, 60, 180, and 600 ppm for 2 weeks	Induction of genes associated with complexes I through IV of the electron transport chain
			Overexpression of genes associated with fatty acid oxidation, Krebs cycle, apoptosis, and membrane transporters

Lu et al. [38]	Wistar rats	100, 200, and 240 mg/kg for 8 days	Increased liver/body weight ratio
			Signs of diffuse hepatocyte degeneration with the highest doses of UA
			Changes in energy, amino acid, lipid, and nucleotide metabolism

Liu et al. [39]	Wistar rats	100 and 240 mg/kg for 10 days	Changes in expression of proteins related to oxidative stress and lipid metabolism

Moreira et al. [40]	Wistar rats	Infusion of liver with solutions 1, 2.5, 5 and 10 *μ*M at an infusion rate of 10 *μ*L/min	Increase of oxygen consumption, decrease of ATP level, and inhibition of gluconeogenesis at lower UA concentration
			Inhibition of mitochondrial electron flow and medium-chain fatty acid oxidation at highest UA concentrations

## Data Availability

The article is a review, and the related articles can be found in literature.
